# The medical and scientific legacy of Juan José Gagliardino

**DOI:** 10.20945/2359-4292-2024-0322

**Published:** 2024-08-09

**Authors:** Licio A. Velloso, Antonio C. Boschero, Bruno Geloneze

**Affiliations:** 1 Universidade de Campinas Centro de Pesquisa em Obesidade e Comorbidades Campinas SP Brasil Centro de Pesquisa em Obesidade e Comorbidades, Universidade de Campinas, Campinas, SP, Brasil

**DEAR EDITOR,**

In May, 2024, the medical and scientific communities of Argentina, Latin America, and the whole world have lost an iconic leader who played a central role in the fields of endocrinology, metabolism, diabetes, and medical education. Juan José Gagliardino, or simply Juanjo, was born in 1938, in the city of la Plata, Argentina. He obtained an MD in 1962, from the Faculty of Medical Sciences in La Plata and a PhD from the same institution, working with pancreatic regeneration. During his productive career, Juanjo published over 600 scientific articles with a special emphasis on the development of strategies to treat patients with diabetes in developing countries, where access to medical care, laboratory testing, and drug therapy is scarce. His leadership in this area was the basis for the International Diabetes Foundation/Eli Lilly 1995 Award entitled Diabetes Education: from health-care team members to patients with diabetes and their relatives. Juanjo was a member of the International Federation of Diabetes and several other national societies devoted to the treatment, education, and research in the field of diabetes. During the final years of his remarkable career, Juanjo devoted much of his time and effort to providing advanced strategies to educate the next generation of doctors. Juanjo was a kind and friendly person who collected admirers all over the world. His academic and scientific achievements will long be remembered as a symbol of determination and commitment to improving the living conditions of vulnerable people who require diabetes treatment.

Some high-impact articles published by Juan José Gagliardino (1-3).

Some articles of Juan José Gagliardino published in collaboration with Brazilian scientists (4-6).
